# Electric eels use high-voltage to track fast-moving prey

**DOI:** 10.1038/ncomms9638

**Published:** 2015-10-20

**Authors:** Kenneth C. Catania

**Affiliations:** 1 Department of Biological Sciences VU Station B, Vanderbilt University, Box 35-1634 Nashville, Tennessee 37235, USA

## Abstract

Electric eels (*Electrophorus electricus*) are legendary for their ability to incapacitate fish, humans, and horses with hundreds of volts of electricity. The function of this output as a weapon has been obvious for centuries but its potential role for electroreception has been overlooked. Here it is shown that electric eels use high-voltage simultaneously as a weapon and for precise and rapid electrolocation of fast-moving prey and conductors. Their speed, accuracy, and high-frequency pulse rate are reminiscent of bats using a ‘terminal feeding buzz' to track insects. Eel's exhibit ‘sensory conflict' when mechanosensory and electrosensory cues are separated, striking first toward mechanosensory cues and later toward conductors. Strikes initiated in the absence of conductors are aborted. In addition to providing new insights into the evolution of strongly electric fish and showing electric eels to be far more sophisticated than previously described, these findings reveal a trait with markedly dichotomous functions.

## Abstract (web summary)

Weakly electric fish species use low-voltage to sense their surroundings, whereas a few strongly electric species use high-voltage as a weapon. Here the author shows that electric eels cross this boundary, using high-voltage simultaneously as a stunning weapon and as an electrical radar system to track fast-moving prey.

## Graphical abstract

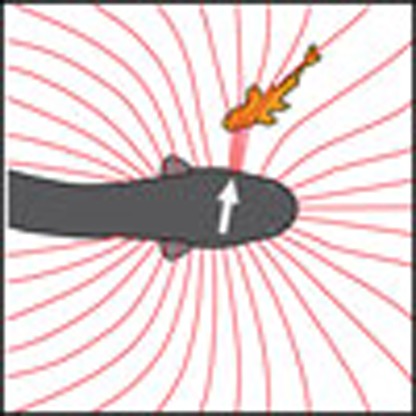

Few species have garnered more historical interest and investigation than electric eels. First used as a prized source of electricity in experiments by Walsh^1^, Humboldt^2^ and Faraday^3^ they later played a pivotal role in the isolation of the acetylcholine receptor^4^ and determining the structure of voltage-gated sodium channels^5^. For centuries it was obvious that eels used their high voltage as a weapon, but how it evolved remained a mystery to Darwin^6^ who considered electric organs under ‘Special Difficulties of the Theory of Natural Selection'. The difficulty in the case of strongly electric fish was that no function had been ascribed to the smaller electric organs that were present in many extant species and that must have been present in eel ancestors. The discovery in the 1950s that weakly electric fish generate minute electric fields as part of an elaborate sensory system^7^ solved this longstanding mystery. Weakly electric fish, including close relatives of the electric eel, are able to discriminate objects with electricity by monitoring distortions in a self-generated electric field surrounding their body^8^,^9^,^10^,^11^. Electric eels retain this low-voltage sensory system^12^,^13^ including a weak electrical discharge and corresponding electroreceptors (Fig. 1). But the possibility that the eel's high-voltage discharge plays a sensory role has been overlooked.

The experiments described below address this question by taking advantage of the binary nature of the eel's hunting behaviour. Eels emit the low-voltage weak output while searching and the high-voltage output when striking. Strikes begin with high-voltage volley onset (≈400 Hz, Fig. 1) followed milliseconds later by rapid head translation, and culminate in suction-feeding when the target is reached. Here experiments show that eels can find and track conductors using these high-voltage volleys without the aid of vision, mechanosensation, chemoreception or biogenic electric fields.

## Results

### Experiments using stationary conductors

**Figure 2:**
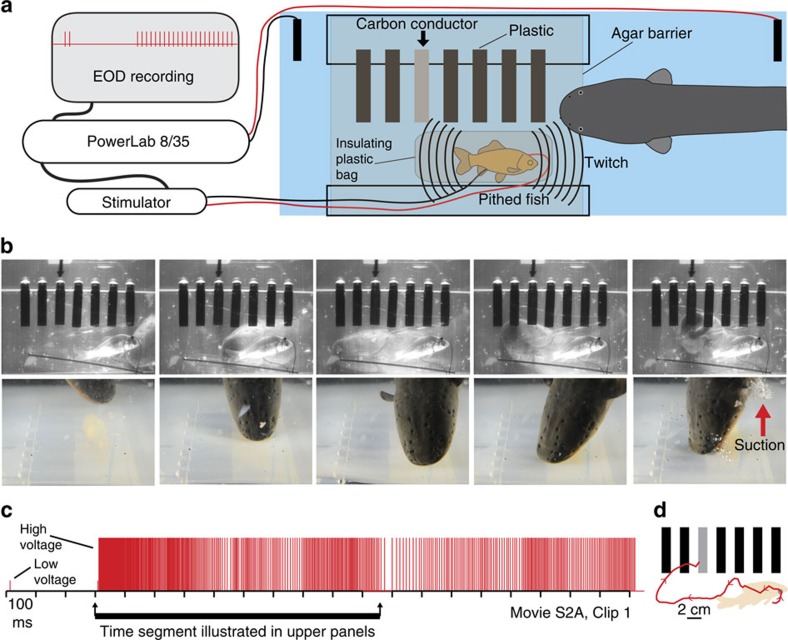
Paradigm showing that electric eels find and attack conductors. (**a**) Recording and stimulator configuration that triggered pithed-fish twitch and eel attack in the presence of six plastic rods and one conductor (arrow). (**b**) Plates from high-speed movie (top) and real-time (bottom) of same trial illustrating circuitous path to conductor. (**c**) Eel low- and high-voltage discharge marked with short, and tall ticks, respectively, illustrating the exclusive use of high-voltage during strike movement. (**d**) Eel path to conductor.

In the first set of experiments eel attacks were elicited by twitch artificially generated from a prey fish. Electric eels use hunting ‘doublets' (two closely spaced high-voltage discharges^14^,^15^) to induce involuntary twitch in nearby hidden prey, which generates a mechanosensory cue detectable by the eel^15^. For these experiments prey fish were anaesthetized, pithed to destroy the brain and electrically insulated in a plastic bag. Twitch in the pithed-fish preparation was generated through a Grass stimulator that could be triggered by the investigator or by a PowerLab unit in response to an eel hunting doublet. The pithed-fish preparation was covered with a thin electrically permeable agar barrier^16^,^17^ that did not mask mechanosensory cues^6^. For one eel (eel A), preliminary data were collected for attacks either in the presence of only the insulated fish preparation, or alternatively in the presence of the fish preparation and a conductive carbon rod. When only the insulated fish preparation was present, the eel responded to fish twitch with a high-voltage volley and rapid strike often towards and over the fish, but the strike never culminated in a suction-feeding attempt (Supplementary Movie 1) characteristic of final prey capture (six trials). When a conductive carbon rod was added to the preparation, fish twitch elicited high-voltage volleys, rapid head translation and a violent suction-feeding attempt (Supplementary Movie 1) at the carbon rod (six trials). These observations were followed by more detailed investigation in two additional eels (Fig. 2). In this second paradigm, the insulated fish preparation was placed next to a series of six equally spaced plastic rods and a single, conductive carbon rod (relocated between trials), all covered with a thin electrically permeable agar barrier (Fig. 2a).

Fish twitch again elicited eel high-voltage attack volleys and rapid strikes. The eel strikes took variable and often indirect paths, but always culminated with an aggressive suction-feeding attempt directed at the conductor (Fig. 2, Supplementary Fig. 1, Supplementary Movie 2A and 2B). The attacks were significantly concentrated on the conductor when considered as a two-choice test between any plastic rod or the single conductive carbon rod (eel B, *n*=6 trials, *P*=0.016; eel C, *n*=12 trials, *P*=0.00024). Eels never attacked the non-conductive plastic rods. It was confirmed for all trials that no low-voltage discharges were emitted during the striking movement and initial suction-feeding attempts (Fig. 2, Supplementary Fig. 1). Evidence of sensory conflict—as indicated by eels first moving towards the mechanosensory stimulus (fish twitch), and then later (often in the opposite direction) towards the conductor, was observed for some trials for each of the 3 eels (for example, Supplementary Movie 1, 2A and 2B, Supplementary Fig. 1). The experiment was repeated for eel C, but using seven non-conductive plastic rods and no conductor. In this paradigm (six trials) the eel emitted high-voltage volleys in each trial and struck out towards the fish preparation, but ignored the plastic rods and aborted the strikes without a suction-feeding attempt to any location (Supplementary Movie 3). The results of these trials clearly show that eels modify their ongoing strike trajectories based on conductor location and often completely reverse direction during the strike. The results also suggest eels do not complete suction-feeding strikes in the absence of a conductor.

### Experiments using moving conductors

To better assess eel accuracy and provide more definitive evidence for the use of the high-voltage output for electrolocation, a single carbon conductor and a number of similarly shaped non-conductive objects were mounted on a spinning plastic disk (Supplementary Fig. 2 and Methods) at the bottom of an aquarium, below an agar barrier, and eel behaviour was recorded at 30 fps using undetectable 940-nm infrared illumination to prevent the use of vision^18^,^19^. Separate 940-nm diodes were triggered to mark either the low-voltage output (one diode) or the high-voltage output (both diodes) for convenient interpretation of the supplementary movies in slow motion. Eel discharges were simultaneously recorded. Three eels were tested in this paradigm (eels A, C, and D). Each eel struck exclusively at the moving conductor, which traversed a curved path and was tracked by striking eels during the high-voltage discharge (Supplementary Fig. 2). Four suction-feeding strikes at the conductor were obtained from eel A and five suction strikes were obtained from each of eels C and D. The concentration of strikes at the conductor was significant for each animal as compared to random attacks to any of the stimuli (*P*=0.0017 eel A; *P*<0.001 for eels C and D see Methods). This behaviour was not maintained beyond four trials for eel A, which received no reward for striking. However after 5 trials eels C and D were rewarded with a fish following each strike. In this manner the striking behaviour was maintained for five additional trials for eel D with the same result.

**Figure 3:**
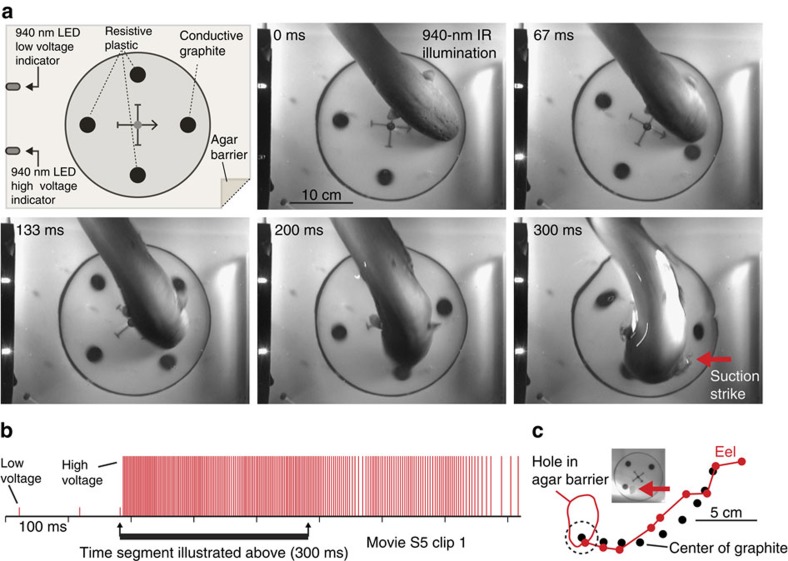
Eel conductor tracking under 940 nm IR illumination. (**a**) Schematic of paradigm and eel tracking behaviour and suction-feeding strike to conductor. (**b**) Eel low and high voltage discharge marked with short, and tall ticks, respectively, illustrating the exclusive use of high-voltage during strike movement. (**c**) Eel track relative to conductor movement. Inset shows hole in agar (arrow) that was directly over conductor at suction onset.

For eel C the experimental paradigm was refined by reducing the conductor to a small, 2.5-cm carbon disk that was embedded in the larger spinning disk, along with three embedded plastic disks as control objects (Fig. 3). This refinement revealed remarkable speed and accuracy of tracking and strikes culminating in suction-feeding attempts (*n*=10, *P*<0.0001 significance as described above, see Methods) at a small, fast-moving conductive object on a curved trajectory (Fig. 3, Supplementary Fig. 3, Supplementary Movies 4 and 5). In addition, in some of the trials the eel reversed direction during the strike as the conductor passed from left to right (Supplementary Fig. 3c). For all of the tracking trials described above, no visual cues were available, only high-voltage discharges occurred during the striking movements, and the agar barrier prevented diffusion of potential chemical cues and likewise prevented mechanosensory cues from direct contact. The use of inert carbon conductors prevented galvanic fields, typical for metal conductors in water, that could be detected with passive electroreception^20^. Finally, visually and mechanically similar plastic objects provided redundant control for mechanosensation and vision and were never attacked.

**Figure 4:**
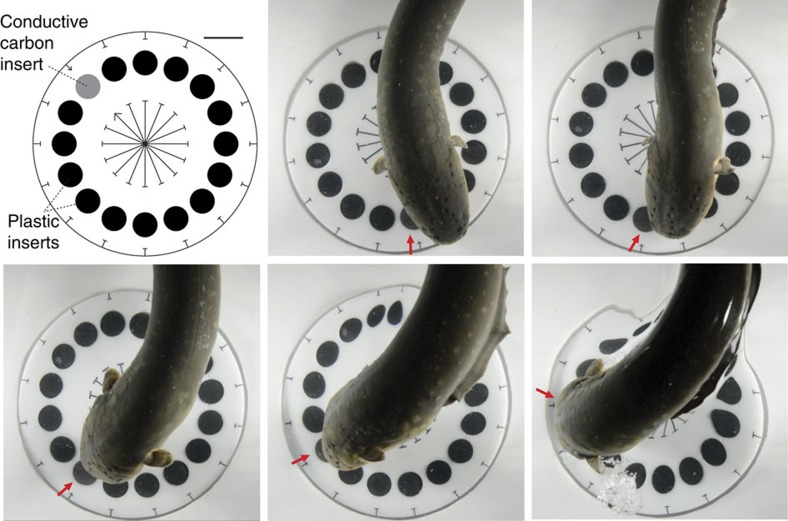
Schematic of paradigm used to illustrate tracking strike under full spectrum illumination along with plates from real-time video. A single conductor was inserted along with 15 plastic non-conductors of similar appearance. For clarity, added red arrow marks the conductor in figure, which was indicated on the disk with a smaller arrow. The eel rapidly accelerated to catch up to the conductive stimulus, then tracked the stimulus as is initiated a suction-feeding strike. For reference, each disk is 2.54 cm wide, centered on a circle of 16.5 cm diameter, spinning at a rate of 0.88 revolutions per second (see Supplementary Movie 6).

The use of low intensity infrared illumination in the former trials precluded high-speed videography. To obtain high-speed movies for eel tracking behaviour under full spectrum illumination while retaining control for vision, the paradigm was further refined by creating a spinning plastic disk containing 15 closely spaced, 2.5-cm diameter plastic inserts and a single conducting carbon insert (Fig. 4). To better gage strike accuracy, agar was not used as a barrier (see Methods). This paradigm showed that an eel can overtake and track a conductor moving at 45 cm s^−1^ with rapid acceleration followed by apparent matching of conductor speed (Supplementary Movie 6). This is faster than previously reported for the use of active electrolocation in weakly electric fish^21^.

**Figure 5:**
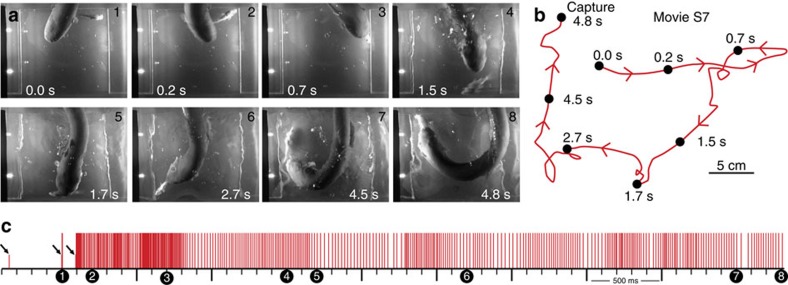
Eel tracking fish below agar barrier under 940-nm infrared illumination. (**a**) Plates from movie with dual, 940-nm diodes indicating high-voltage output. (**b**) Eel track with plates marked (circles). (**c**) Eel low- and high-voltage discharge marked with short, and tall ticks, respectively, illustrating the exclusive use of high-voltage during strike movement. Circles mark timing of plates in **a**. Scale bar, 4 cm.

Finally, eels readily struck at prey fish, below an agar barrier, under 940-nm illumination. Numerous trials revealed accurate high-speed tracking of live fish moving along non-linear paths, similar to the conductor tracking described above, exclusively during the high-voltage discharge (Fig. 5, Supplementary Movie 7).

## Discussion

The results show the electric eel's high-voltage discharge is not just a weapon; it is also part of a sophisticated tracking system for guiding strikes at fast-moving prey. During most strikes the high-voltage discharge briefly arrests all voluntary behaviour, cancelling escape responses and providing a critical advantage to eels^15^. But it does not prevent rapid ongoing movement through the water (for example, Supplementary Movie 8). As a result the eel's strikes must often be guided by continual sensory feedback to reach the target. Sensory feedback from the high-voltage discharge is integral to the eel's attack, given that strikes were aborted in the absence of conductors. Aborted strikes in the absence of a conductor are also evident in Supplementary Data from a previous study, but their significance was not appreciated (see Supplementary Movie 6 in ref. 15).

The use of the high-voltage, high-frequency discharge to guide the strike has obvious parallels with bats producing a terminal feeding buzz of echolocation calls during the close range, final attack on flying insects^22^. In both cases high-temporal resolution is required for accuracy and in both cases this is achieved by increasing the rate of the probing output. Some weakly electric fish have also been shown to increase their pulse discharge rate when investigating objects^23^,^24^ and for mormyrids hunting cichlids in lake Malawi this has similarly been likened to bat behaviour^25^. For electric eels, the use of high voltage presumably also increases the range of active electroreception compared to their low-voltage output^26^. This could explain why water disturbances often the trigger high-voltage volleys accompanied by an explosive strike. High-voltage onset and head translation toward a water disturbance likely allows the eel to ‘acquire' the conductive prey with its longer range, high-temporal resolution electrosensory system. This suggestion is supported by the artificial separation of mechanosensory and conductance cues during experiments (Fig. 2). Eels started towards the water movement, but used active electrosensory feedback to guide the final strike towards the conductor. Although the separation of cues was artificial in the laboratory, a water movement cue in nature could emanate most strongly from the former position of an escaping fish^27^ making electrolocation the most accurate sensory modality for guiding pursuit. And unlike mechanoreception, active electroreception is presumably unaffected by the eel's own movement through the water. Finally, the ability to simultaneously immobilize and track prey with high voltage is an unusual combination of dichotomous functions. These results cast the electric eel in a new light, as both a formidable predator and unique sensory specialist.

## Methods

### Animals

All procedures were approved by the Vanderbilt Institutional Animal Care and Use Committee. Four eels (Electrophorus electricus) were purchased from a commercial fish supplier and housed individually in custom-made Plexiglas aquariums ranging in size from 80 to 120 gallons (300–480 l) with aerated water, gravel bottom, rocks, plastic imitation branches, and plastic plants with water temperature maintained between 24 and 28 °C with thermostatically controlled aquarium heaters and pH between 6.5 and 7.5. Lighting was on a 12/12 light–dark cycle and eels were fed earthworms, fish and crayfish. Eels ranged in size from 55 cm (one specimen; eel D) to 70–80 cm (two specimens; eels B and C) and 115 cm (one specimen, eel A).

### Recordings of eel behaviour

For recordings of eel behaviour animals were transferred to either a 75 × 30 × 12 cm or 150 × 56 × 25 cm (L × W × H) custom-made Plexiglas aquarium. Water temperature was 25–26 °C, pH 7.1–7.4. Conductivity was maintained between 125 and 200 μS cm^−1^. The electric organ discharges were recorded using carbon electrodes (1.4 × 30 cm) in the water connected on their exposed tips to wire leads from a split BNC cable that connected directly to one channel of either a PowerLab 8/35 or PowerLab 4/30 data acquisition unit (ADInstruments) sampling at a minimum of 100 K per second and in turn connected to a MacPro laptop running LabChart 7 software (ADInstruments). High-speed video was collected with a MotionPro HS-3 camera (Redlake) at 1,000 frames per second with two RPS Studio CooLED 100 RS-5610 for lighting at 904-ms shutter speed using the circular recording mode for capturing events. The camera's synchronization output was recorded on a separate PowerLab channel allowing precise coordination of each frame with other recorded events (for example, the EOD). Video was transferred to a MacPro laptop using MotionProX software (Integrated Tools Design). Concurrent real-time colour video was collected with either a Nikon D4 SLR (Nikon Inc, Melville, NY) set to video mode, or a Flip video camera (Cisco Systems Inc.). To illustrate the relationship of each high-voltage EOD to behaviour in the Supplementary Movies, each frame during which an EOD occurred (at 1,000 fps each EOD peak corresponded to a unique frame) was colourized in Photoshop CS 6 (Adobe Systems Incorporated). The tiff format image files were then opened in sequence in QuickTime Player 7 Pro (Apple Inc) and the sequence was exported as a QuickTime movie. For infrared recording a low-light CCD camera (KT&C security camera) was use while illuminating the scene with two IR-Flood Ultra-Covert 940 nm illuminators (Night Vision Experts, Buffalo, NY, USA).

### Stationary conductor trials

To generate a prey fish twitch that elicited an eel attack (Fig. 2a), prey fish were anaesthetized with 2% buffered MS222 and pithed to destroy the brain. Leads of an SD9 stimulator were connected through the fish pithing hole and the fish rectum. The output of the PowerLab was in turn connected to the triggering input of an SD9 Grass stimulator set for output of 5 volts, 1 ms and no delay. The fish was then placed into a small Ziploc bag that was sealed around the electrodes. Trials during which the stimulator was active but attached to a freeze-thawed, pithed-fish (which did not twitch) confirmed the mechanosensory nature of the response, as no responses were obtained from electrodes in the absence of twitch (10 trails each eels A, B, and C). Twitch was generated either arbitrarily by the investigator triggering the SD9 stimulator (all 6 trials Eel B, 6 of 12 trials eel C), or the ‘Fast Response Output' feature of LabChart was used to trigger the stimulator in response to an eel hunting doublet (6 of 12 trials eel C). The pithed-fish preparation in the plastic bag was placed below and agar barrier (1% agar 6–10 mm thickness) adjacent to six plastic rods and one carbon rod (McMaster-Carr, Atlanta, GA). To illustrate the high and low voltage output of the eel during each strike, the EOD recording was copied at high-resolution from the LabChart program into Adobe Illustrator and each eel output was marked with small and large tick-marks for low and high voltage, respectively. Significance in the bar-choice task was conservatively assessed as a two choice test (binomial test) between any plastic rod or the single conductor (no plastic rods were attacked in the course of the study). A conservative two choice test was used because in some trials the eel was distant from a number of the plastic control rods at high-voltage onset, making it unlikely that all six of the rods would be part of the electrosensory scene.

### Moving conductor trials

To investigate the response of electric eels to moving conductors, eels B and D were tested using 5 plastic rods of 8 cm length and 1.3 cm diameter, and a single conductive rod of the same length and diameter, all glued to the surface of a 13-mm thick, 16.5 cm diameter white Plexiglas disk (Supplementary Fig. 2). Eel A (the largest eel) was tested with two trials as described above, but also with two trials using 3, 1 in diameter plastic rods and a single 1 inch diameter carbon rod as conductive target. A 1.3-cm hole was drilled in the center of the disk, for placement of the disk on a short axel at the bottom of the aquarium. The disk with conductors was made to rotate either with a string coiled around an extension at the base of the disk that was pulled manually (all ‘rod' trials eel D) or by use of magnets embedded in the base of the disk, and a second set of magnets attached to a rotating drill below the aquarium (all ‘rod' trials eels A and C). No difference in eel response was noted for the two different methods of disk rotation. The disk and conductors were then covered with an agar barrier as described above. Several small air bubbles were intentionally introduced into the cooling agar so it was more readily visible in the supplementary movies. Although eels made numerous orienting responses towards the conductor during both low-voltage and high-voltage discharges, only suction-feeding attempts characterized by air release from the operculum were scored as attacks. High-voltage tracking was considered to have occurred if the striking movement was made during the high-voltage discharge (as was the case for all trials) and there was no overlap in the final strike location at the conductor, and the initial position of the conductor at high-voltage onset. Significance was assessed based on the null hypothesis of random attack to any of the rods (for example, 1 in 6 probability when using 5 plastic rods and 1 conductor). For tracking smaller conductors (Fig. 3, Supplementary Fig. 3) four, 2.54 cm diameter holes were drilled through a 16.5-cm disk at 90 degree intervals, centered on an 8.3 cm radius. Three, 2.54-cm diameter plastic disks were inserted flush to the surface, and a single 2.54 conductive carbon disk was inserted flush to the surface (Fig. 3). The same method was used, but with more plastic disks, for the high-speed video trials (Fig. 4). The conductor was marked with a central arrow for reference on the movie. The disk was rotated either manually with string as described above (Supplementary Movie 5, Clips 1–5; Supplementary Movie 6) or with magnets as described above (Supplementary Movie 4, Supplementary Movie 5, Clip 6). Agar covered the spinning disk in the infrared video trials. An agar barrier was not used for the high-speed trials, because eel suction-feeding behaviour prematurely arrested tracking when the eel grasped the agar in its mouth. This was insignificant for the widely spaced stimuli in infrared trials, but made strike accuracy harder to interpret with closely spaced stimuli.

## Additional information

**How to cite this article:** Catania, K. C. Electric eels use high-voltage to track fast-moving prey. *Nat. Commun.* 6:8638 doi: 10.1038/ncomms9638 (2015).

## Supplementary Material

Supplementary Information Supplementary Figures 1-3

Supplementary Movie 1 Preliminary trials showing effect of conductor on eel strike behavior. In the first trial, the eel responds to fish twitch in the absence of a conductor and does not make a final suction feeding attempt. When a conductor is present, the eel changes course mid-strike and attacks the conductor.

Supplementary Movie 2a Trials showing an electric eel finding a conductor during the high voltage discharge. Pithed fish twitch is generated with a stimulator through electrodes to eliciting eel attack. Strikes take variable paths but culminate in an aggressive attack aimed at the conductor. The eel often reverses direction during the strikes. Trials are show first in slow motion, and then in real time. Red frames in the slow motion indicate the timing of each high voltage discharge. Note that for some trials (Clips 1 and 2) the fish twitch was triggered by the experimenter, whereas for other trials (Clips 3) the stimulator was configured to cause twitch in response to eel doublet.

Supplementary Movie 2b Additional trials showing an electric eel finding a conductor during the high voltage discharge. Pithed fish twitch is generated with a stimulator through electrodes to eliciting eel attack. Strikes take variable paths but culminate in an aggressive attack aimed at the conductor. Trials are show first in slow motion, and then in real time. Red frames in the slow motion indicate the timing of each high voltage discharge. Note that for some trials (Clip 4) the fish twitch was triggered by the experimenter, whereas for other trials (Clips 5 and 6) the stimulator was configured to cause twitch in response to eel doublet.

Supplementary Movie 3 Electric eel responding to fish twitch (triggered through stimulator by eel doublet) in the absence of a conductor - all 7 stimuli are plastic rods. In contrast to trials with a conductor present, here the eel aborts strikes without a suction feeding attempt.

Supplementary Movie 4 Eel tracking a conductor under 940 nm IR illumination. Eel strikes at conductor (center arrow) located below an agar barrier. Paradigm includes redundant control for vision with plastic inserts of similar appearance. The clip is shown in slow motion with sound, and shows two full strikes during high-voltage discharge and several partial strikes (not scored).

Supplementary Movie 5 Eel strikes at a fast moving conductor (as in movie S4) located below an agar barrier with 940nm IR illumination and redundant control for vision with plastic inserts of similar appearance. These clips are in real-time.

Supplementary Movie 6 Eel pursuit and attack of a conductor under full spectrum illumination to allow high-speed videography. This trial is shown first in real-time, and then in slow motion at 1000 frames per second. Green flashes indicate the low-voltage output, red flashes indicate the high-voltage output.

Supplementary Movie 7 Eel tracking and capturing a fish below and an agar barrier under 940nm IR illumination. Note the trial begins with a fish-stimulating doublet, which causes an escape response, followed by eel attack. The fish is periodically immobilized, but the agar barrier delays capture. The eel makes several holes in the agar, and ultimately catches the fish. This predatory sequence showing prey tracking occurs during the high-voltage discharge and is consistent with a general strategy of using conductance to identify and track prey during the strike.

Supplementary Movie 8 Fish capture sequence in real time. This trial illustrates why eels need to track prey despite being able to inactive prey voluntary movement with the high-voltage discharge. Note also the eel's fast reversal to capture the prey during the high-voltage discharge. This latter behavior is common in weakly electric fish during electrosensory guided prey capture.

## Figures and Tables

**Figure 1:**
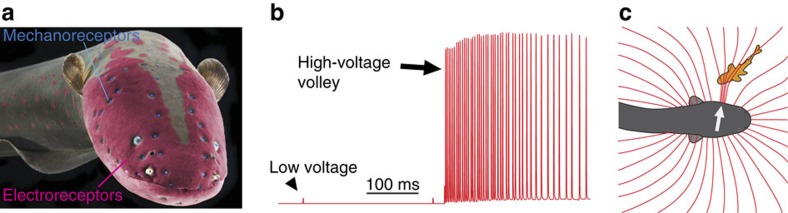
Electric eel senses and discharge. (**a**) Eel colourized to show electroreceptors (magenta) and mechanoreceptors-neuromast canals (blue). (**b**) The weak, low-voltage output used for electrolocation and the high-voltage, high-frequency output used as a weapon. (**c**) Schematic illustration of electrolocation based on the convergence of electric field lines on the eel's skin (arrow).
