# Supplementary material for: Electric eels use high-voltage to track fast-moving prey
Source: Nat Commun. 2015 Oct 20;6:8638. doi: 10.1038/ncomms9638 (PMC4667699; doi:10.1038/ncomms9638)
Supplement: Supplementary Information — Supplementary Figures 1-3 [file ncomms9638-s1.pdf]

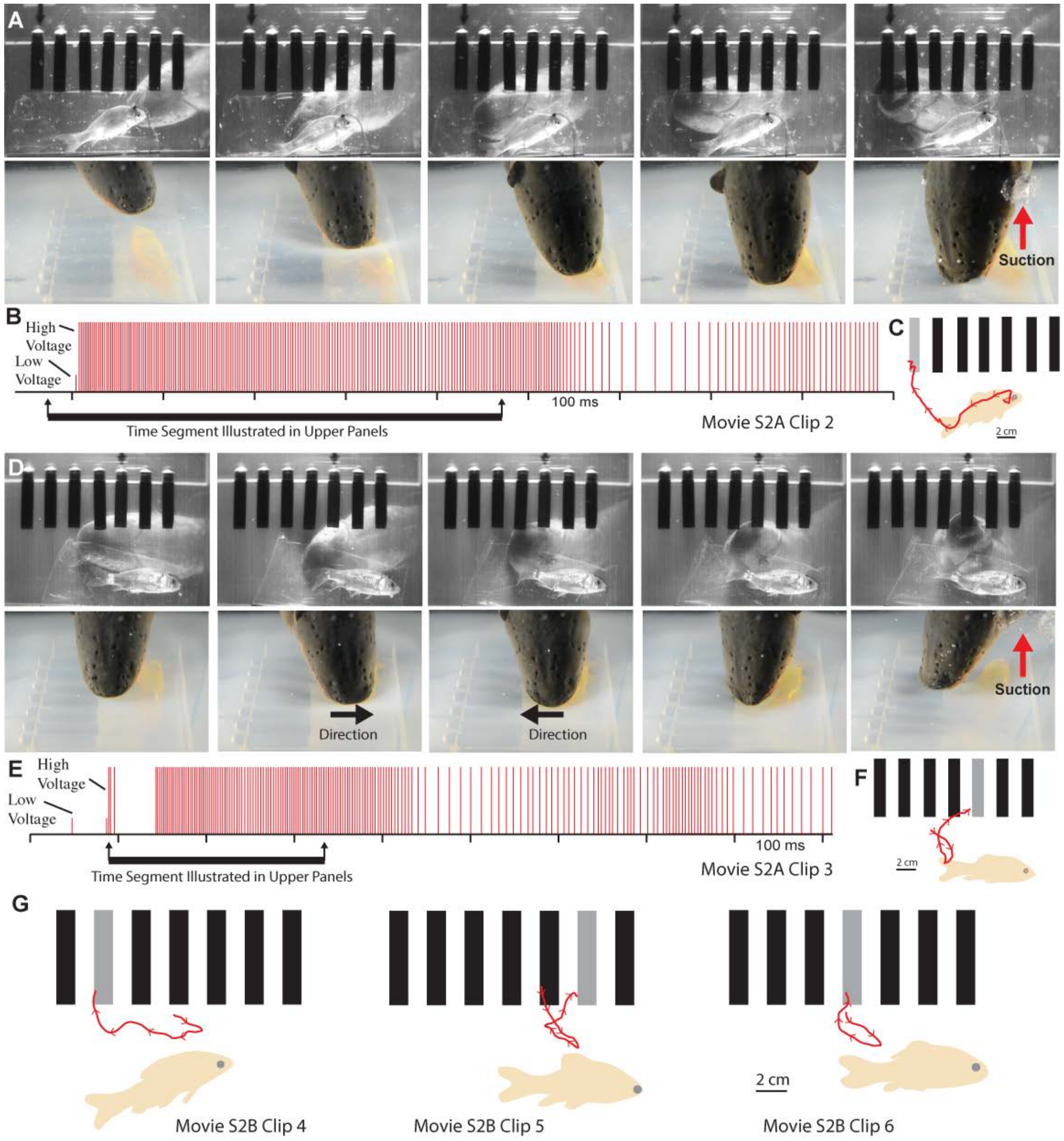

Supplementary Figure 1 Additional trials showing that electric eels find and attack conductors during the high-voltage discharge. A. Plates from high-speed video (top) and real-time (bottom) of same trial illustrating path to conductor. Suction attacks are evidence when air is released through operculum (red arrow) B. Eel low and high voltage discharge marked with short, and tall ticks respectively, illustrating the exclusive use of high-voltage during strike movement. C. Eel path to conductor. See Movie S2, Clip 2 for this trial. D-F conventions as show above. See Movie S2, Clip 3 for this trial. The eel reverses direction during the strike in this trial (F). G. Additional eel tracks from additional trials. The eel reverses direction in right 2 panels. See Movie S2 for these and additional trials in slow motion and real time. Note that in A-C, the experimenter triggered fish twitch resulting in eel attack. In D-F, the fish twitch was triggered (through the stimulator) by the eel's doublet – see methods.

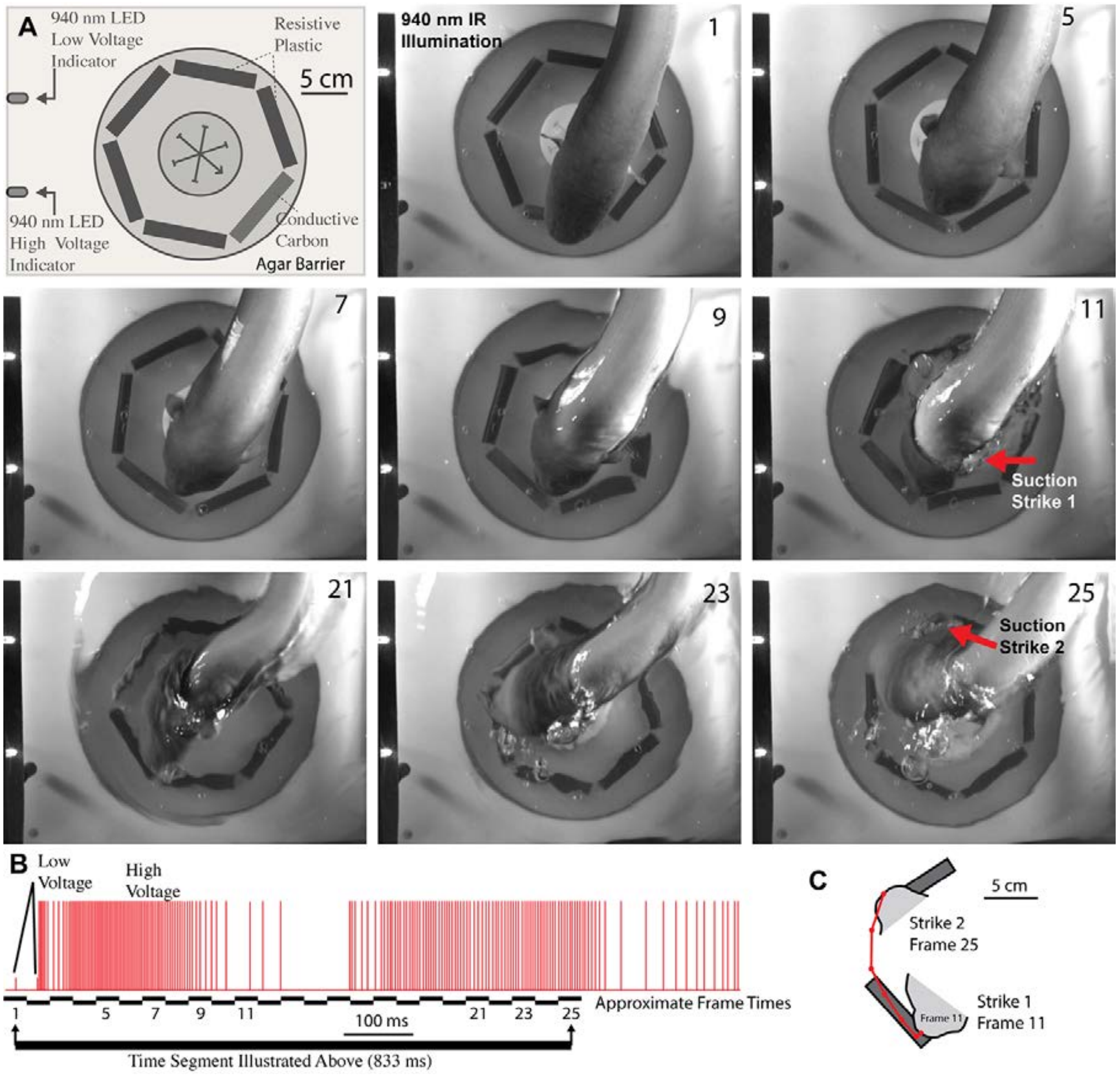

Supplementary Figure 2 Additional trial showing eel conductor tracking under 940 nm IR illumination. A. Schematic of paradigm using 5 plastic rods and a single conductive carbon rod (all under agar barrier) and plates illustrating eel attack behavior. B. Eel low and high voltage discharge marked with short, and tall ticks respectively, illustrating the exclusive use of high-voltage during strike movement. C. Front of eel illustrated for each of two strikes, and corresponding track during high-voltage discharge.

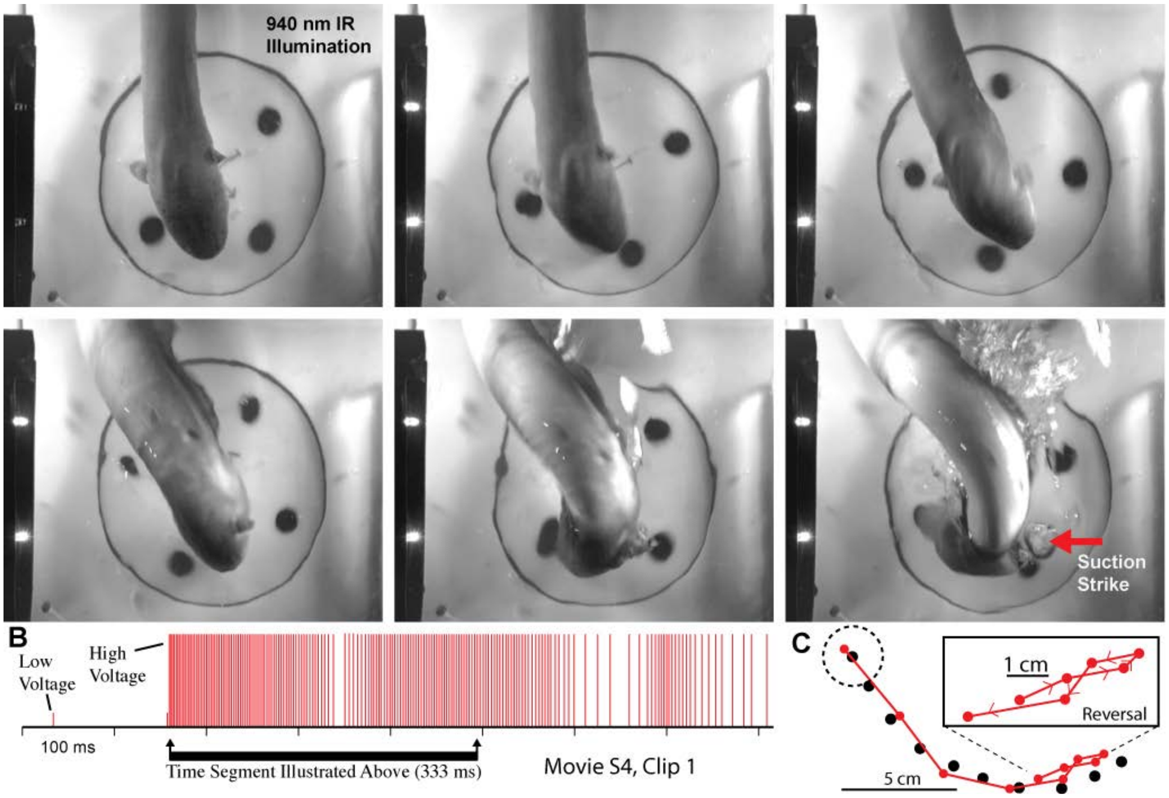

Supplementary Figure 3 Eel tracking a small conductor, under an agar barrier, with 940 nm IR illumination. A. Plates from video showing eel rapid tracking behavior. Dual 940nm diodes (left) indicate high-voltage discharge. Note reversal of eel strike direction as conductor passes from left to right under eel. B. Eel low and high voltage discharge marked with short, and tall ticks respectively, illustrating the exclusive use of high-voltage during strike movement. C. Eel track relative to conductor movement (black dots are center of conductor). Inset shows strike reversal. Disk is rotating at 1 revolution per second. See Movie S4, Clip 1 for this trial in slow motion.
